# Predictive Factors of Patient Satisfaction with Pharmacy Services in South Korea: A Cross-Sectional Study of National Level Data

**DOI:** 10.1371/journal.pone.0142269

**Published:** 2015-11-05

**Authors:** Sunkyung Lee, Onyeka Peter Godwin, Kyungah Kim, Euni Lee

**Affiliations:** 1 Center for Minority Health Services Research, College of Pharmacy, Howard University, Washington, D. C., United States of America; 2 Department of Clinical and Administrative Pharmacy Sciences, School of Pharmacy, Chapman University, Irvine, California, United States of America; 3 The Degge Group Ltd., Fairfax, Virginia, United States of America; 4 Seoul National University College of Pharmacy, Seoul, South Korea; The University of Hong Kong, HONG KONG

## Abstract

**Objectives:**

Patient satisfaction has emerged as a prerequisite to improving patients’ health behaviors leading to better health care outcomes. This study was to identify predictive determinants for patient satisfaction with pharmacy services using national-level data.

**Methods:**

A cross-sectional evaluation was conducted using 2008 Korean National Health and Nutrition Examination Survey (KNHANES) data. To assess the predictive factors for patient satisfaction with pharmacy services, an ordinal logistic regression model was conducted adjusting for patient characteristics, clinical comorbidities, and perception of health.

**Results:**

A total of 9,744 people, a representative sample of 48.2 million Koreans, participated in the 2008 KNHANES, of whom 2,188 (23.6%) reported visits to pharmacy within the last 2 weeks prior to the survey. Of the patients who visited the pharmacy, 74.6% reported to be either “very satisfied” or “satisfied,” and 25.4% responded as being “neutral,” “dissatisfied,” or “very dissatisfied.” A multivariate ordinal logistic regression analysis with weighted observations revealed that patients with fair perception of health (adjusted OR 1.32; 95% CI 1.01–1.74; *p*<0.05) and those with middle to low family incomes (adjusted OR 1.34; 95% CI 1.02–1.76; *p*<0.05) were more likely to be satisfied with pharmacy services, and employment-based insurers were less likely to be satisfied with pharmacy services (adjusted OR 0.80; 95% CI 0.65–0.97; *p*<0.05).

**Conclusion:**

Our findings indicated that three out of four patients expressed satisfaction toward pharmacy services. Middle to low family incomes, fair perception of health, and employee insured individuals were significant predictors of patient satisfaction with pharmacy services.

## Introduction

Pharmacy practice has considerably expanded to provide patient-centered care [1]. Consequently, efforts in evaluating patient satisfaction and the development of instruments measuring the outcomes have been documented from various countries in the literature [2–6]. Patient satisfaction is an important humanistic outcome as a patient’s subjective assessment of health care services [7, 8]. Growing interest in patient satisfaction initially arose as a result of consumerism. However, patient satisfaction has recently emerged as a prerequisite to providing adequate health care, as it may be able to improve patients’ health behaviors, resulting in better health care outcomes [9]. Previous studies demonstrated that patient satisfaction related to medication was potentially important to patient-reported outcome linked to better adherence, which has been a major concern in health care [10–13]. These studies suggested that greater treatment satisfaction of the patients was associated with better medication adherence and improved persistence, and with lower regimen complexity or treatment burden.

South Korea has a unique National Health Insurance (NHI) system with universal coverage covering almost the entire population with a centrally financed national health service [14]. The Ministry for Health, Welfare and Family Affairs (MIHWFA) has overall responsibility and a supervisory role for the health of the population. The National Health Insurance Corporation (NHIC) is the single insurer, responsible for providing health care benefits to the population, as well as collecting contributions and reimbursing providers. In 2000, the separation reform of drug prescribing and dispensing responsibilities between physicians and pharmacists was enforced to provide the general public with better quality of medical services and to prevent the misuse and overuse of medicines [14]. Since the passage of the Korean Health Care System Reform Act of 2000, the overall demand for care services in the pharmacy setting has been reported to rise because of a rapid increase in prescription volume and expenditure [15]. While patient satisfaction with pharmacy services is one of the most important topics for pharmacists in South Korea, very few studies are reported [16] and even fewer studies used national level data. Statistics Korea (KOSTAT), a central government organization for statistics, reported rates of patient satisfaction with pharmacy services on non-prescription medications, it does not explain the overall satisfaction of patients with comprehensive areas of pharmacy services including prescription medications [17]. Therefore, the objective of this study was to evaluate overall patient satisfaction with comprehensive areas of pharmacy services and its predictors in South Korea using the latest national level survey including pharmacy satisfaction.

## Materials and Methods

### Data source and study population

The Korean National Health and Nutrition Examination Survey (KNHANES) is a nationwide, population-based, cross-sectional survey to examine the general health and nutritional status of Koreans, conducted by the Korean Ministry of Health and Welfare and Korea Centers for Disease Control and Prevention (KCDC) since 1998 [18]. This survey includes approximately 10 000 individuals aged 1 year and over in all 192 primary sample units (PSUs) each year as a survey sample and collects information on socioeconomic status, health-related behaviors, quality of life, healthcare utilization, anthropometric measures, biochemical and clinical profiles for non-communicable diseases and dietary intakes with three component surveys: health interview, health examination and nutrition survey. The data from health interview and health examination are collected by trained staff members, including physicians, medical technicians and health interviewers, at a mobile examination center, and dieticians’ visits to the homes of the study participants [18]. The data from patient satisfaction with pharmacy services were collected during twelve pharmacy visits with the 5-level Likert scale: very satisfied; satisfied; neutral; dissatisfied; and very dissatisfied. The participants were asked what the reason is, how many times for pharmacy visits within two weeks, and how much you were satisfied with pharmacy services [18]. KNHANES IV survey data were collected from July 2007 to December 2009, using a complex, stratified and multistage probability-cluster sampling design representing non-institutionalized civilians in South Korea. All statistics of this survey have been calculated using sample weights assigned to sample participants provided by KNHANES in order to reflect a national estimate allowing the results to be generalizable to the entire population in Korea. Our study used KNHANES IV data from 2008, because they specifically included data on patient satisfaction with pharmacy services. As the information is no longer collected in upcoming KNHANES, the 2008 survey was the latest available data source that allowed evaluation at present [18]. This study focused on all adults aged 19 years or older who had reported one or more pharmacy visit(s) within the last 2 weeks prior to the survey collection [18]. The study used a publicly available data from South Korea that does not contain patient identifiers or linkable information.

This study was approved by both the Institutional Review Board of Seoul National University in Korea and Howard University in the United States.

### Patient health status evaluation

The patient’s physical health status was assessed by calculating the total number of chronic diseases. The total number of chronic diseases included cancer, diabetes mellitus, thyroid disorder, cataract, glaucoma, hypertension, hyperlipidemia, stroke, myocardial infarction, angina pectoris, asthma, chronic obstructive pulmonary disease, hepatitis B, atopic dermatitis, anemia, osteoarthritis, rheumatoid arthritis, osteoporosis, and renal failure.

Patients’ perceived health status data were included as a subjective health status measure based on the self-report from the 2008 KNHANES [18]. KNHANES IV collected information on general health status through participants’ self-report question as “How do you assess your own health status?” in responses including 5 levels: “Very good,” “Good,” “Fair,” “Bad” and “Very bad.” Self perceived health status used in this study was recorded into three levels (“Very good/Good,” “Fair,” “Bad/Very Bad”) for analytic purposes according to response to the question.

### Pharmacy satisfaction

The patient satisfaction data were collected during health interview survey [18]. The patient satisfaction component of the KNHANES survey was designed to assess the overall satisfaction with pharmacy services from up to 12 pharmacy visits during a 2-week period [18]. The overall satisfaction on each visit was assessed on a 5-level Likert scale: 1, very satisfied; 2, satisfied; 3, neutral; 4, dissatisfied; and 5, very dissatisfied [18]. For analytic purposes, the study calculated the medians of the patients’ satisfaction scores during 12 pharmacy visits per each person.

### Statistical analysis

The univariate analyses were conducted to describe baseline demographic characteristics for each study population. We calculated the median score of patient satisfaction with pharmacy services due to an ordinal nature of the outcome variable (very satisfied = 1, satisfied = 2, neutral = 3, dissatisfied = 4, and very dissatisfied = 5) as a dependant variable.

Bivariate correlations of covariates, including the clinical comorbidity score and the perception of health, with the level of patient satisfaction on pharmacy services were tested using the chi-squared (^2^) test, and unadjusted and adjusted odds ratios (ORs) were calculated.

To assess the predictive factors for patient satisfaction with pharmacy services, an ordinal logistic regression model was utilized as the dependent variable to determine unadjusted ORs and adjusted ORs with 95% confidence intervals (CIs). The covariates included age, gender, health insurance, urban/rural area, house income, level of education, physical restriction, total number of chronic diseases, and perceived health status. All analyses in the study used weighted data assigned with sample weights of sample participants provided by KNHANES when applicable, to yield national-level estimates, thus reflecting national-level population data. All analyses were performed using SPSS English version 22 Complex Samples Module (IBM, SPSS Statistics 22, Armonk, NY, USA), and SAS 9.2 (SAS institute, Cary, NC, USA)to account for the complex (multistage/stratified/clustered) sampling designs, and to correctly estimate the standard errors with weighted data with 95% CIs and an alpha significant level of less than 0.05.

## Results

A total of 9,744 people, representing 48.2 million South Koreans, participated in the 2008 KNHANES IV survey, of whom 2,188 (23.6%) reported their pharmacy visits among those who aged 19 years and older. Sociodemographic characteristics and the health status of patients with one or more pharmacy visits are summarized in Table 1. Of the patients who visited pharmacies, 74.6%reported to be either ‘very satisfied’ (17.2%) or ‘satisfied’(57.4%), and 25.4% responded as being ‘neutral’ (23.3%), ‘dissatisfied’ (1.8%), or ‘very dissatisfied’ (0.3%). Patients aged between 30 and 39 years had the highest number of pharmacy visits (22.2%), whereas those in the age group of 50–59 years had the least number of visits (17.3%). The number of pharmacy visits made by female patients was similar to those by males (50.6% and 49.4%, respectively). The pharmacy visits were higher in urban areas (80.3%) than rural areas (19.7%).

**Table 1:** Descriptive Characteristics and Health Status of Pharmacy Users from 2008 KNHANES.

Characteristics		Weighted N ^a^ (%)	Un-weightedN(%)
**Total**		11.3 (100.0)	2,188(100.0)
**Patient Satisfaction**	Very Satisfied	1.9 (17.2)	411 (18.8)
	Satisfied	6.5 (57.4)	1,234 (56.4)
	Neutral	2.6 (23.3)	500 (22.9)
	Dissatisfied	0.2 (1.8)	36 (1.6)
	Very Dissatisfied	0.03 (0.3)	7 (0.3)
**Age (years)**	19–29	2.3 (20.3)	279 (12.8)
	30–39	2.5 (22.2)	443 (20.2)
	40–49	2.3 (20.4)	389 (17.8)
	50–59	2.0 (17.3)	380 (17.4)
	≥ 60	2.2 (19.8)	697 (31.9)
**Gender**	Male	5.6 (49.4)	920 (42.0)
	Female	5.7 (50.6)	1,268 (58.0)
**Urban/Rural area**	Urban area	9.1 (80.3)	1,564 (71.5)
	Rural area	2.2 (19.7)	624 (28.5)
**Family Income**	Low	1.7 (15.8)	458 (21.7)
	Mid-Low	2.9 (27.1)	563 (26.7)
	Mid-High	3.2 (29.0)	551 (26.1)
	High	3.1 (28.1)	539 (25.5)
**Education**	Elementary school graduate or less	2.4 (21.6)	655 (31.6)
	Middle school graduate	1.2 (10.3)	233 (11.2)
	High school graduate	4.3 (38.5)	676 (32.6)
	College graduate or higher	3.3 (29.6)	511 (24.6)
**Health Insurance** ^b^	Self-employed (community)	4.5 (40.2)	888 (40.6)
	Workplace (employment)	6.4 (56.3)	1,193 (54.5)
	Others ^c^	0.4 (3.5)	107 (4.9)
**Perceived Health Status**	Very good/Good	4.8 (42.4)	859 (41.3)
	Fair	4.1 (35.9)	677 (32.6)
	Bad/Very Bad	2.4 (21.7)	543 (26.1)
**Physical Restriction**	Yes	1.9 (16.8)	449 (21.6)
	No	9.4 (83.2)	1,629 (78.4)
**Total number of chronic diseases**	No chronic diseases	6.5 (57.2)	1,013 (48.7)
	≥1chronic diseases	4.8 (42.8)	1,068 (51.3)

^a^Weighted frequency of pharmacy users in millions for KNHANES.

^b^Health Insurance: Only accounted for national health insurance.

^c^‘Others’ includes Medical Aid class 1 and 2, no health insurance, or unknown.

In bivariate analysis, family income, health insurance status, and perceived health status were significantly associated with patient satisfaction with pharmacy services (*p*<0.05, data not shown in table).

A multivariate ordinal logistic regression was performed, with sociodemographic characteristics, perceived health status, and clinical conditions as covariates predicting patient satisfaction with pharmacy services (Table 2). Our findings showed that patients with middle to low family incomes were more likely to be satisfied with pharmacy services, compared to those with high family incomes (adjusted OR 1.34; 95% CI 1.02–1.76; *p*<0.05) (Table 2). In particular, a statistically significant association between perceived health status and satisfaction was observed—patients with ‘Fair ‘ perceived health reported higher satisfaction, compared to patients with ‘Very good/Good’ perceived health (adjusted OR 1.32; 95% CI 1.01–1.74; *p*<0.05). In addition, individuals insured through their workplace were less likely to be satisfied with pharmacy services compared to those who were covered through their community (adjusted OR 0.80; 95% CI 0.65–0.97; *p*<0.05). No statistically significant associations were observed between patient satisfaction and education level, age, or gender.

**Table 2:** Unadjusted and Adjusted Odds Ratios and 95% Confidence Interval from Ordinal Logistic Regression on Patient Satisfaction.

Population Characteristics		Patient satisfactionUnadjusted OR(95% CI) ^a^	Patient satisfactionAdjusted OR(95% CI) ^a^
**Age**	19–29	Reference	Reference
	30–39	1.09 (0.78–1.53)	1.06(0.75–1.51)
	40–49	1.00 (0.73–1.36)	1.00 (0.72–1.38)
	50–59	0.88 (0.62–1.25)	0.88 (0.58–1.32)
	≥60	1.05 (0.76–1.45)	1.06 (0.70–1.60)
**Gender**	Female	Reference	Reference
	Male	0.99 (0.81–1.21)	0.99 (0.81–1.22)
**Education**	College graduate or more	Reference	Reference
	High school graduate	0.87 (0.66–1.14)	0.78 (0.58–1.05)
	Middle school graduate	0.77 (0.54–1.09)	0.68 (0.45–1.02)
	Elementary school graduate or less	0.97 (0.74–1.27)	0.86 (0.59–1.25)
**Urban/Rural area**	Urban area	Reference	Reference
	Rural area	1.17 (0.88–1.55)	1.21 (0.90–1.61)
**Family Income**	High	Reference	Reference
	Mid-High	1.16 (0.89–1.52)	1.13 (0.86–1.49)
	Mid-Low	1.35 (1.05–1.74)*	1.34 (1.02–1.76)*
	Low	1.09 (0.79–1.50)	0.99 (0.68–1.43)
**Health Insurance** ^b^	Self-employed (community)	Reference	Reference
	Workplace (employment)	0.82 (0.67–0.99)*	0.80 (0.65–0.97)*
	Others ^c^	1.03 (0.63–1.69)	1.00 (0.61–1.64)
**Physical Restriction**	No	Reference	Reference
	Yes	1.14 (0.90–1.45)	1.17 (0.86–1.59)
**Perceived Health Status**	Very good/Good	Reference	Reference
	Fair	1.33 (1.02–1.73)*	1.32 (1.01–1.74)*
	Bad/Very Bad	1.21 (0.95–1.55)	1.17 (0.88–1.55)
**Total number of chronic diseases**	No chronic diseases	Reference	Reference
	≥1chronic diseases	0.95 (0.78–1.17)	0.91 (0.72–1.16)

^a^Odds Ratios (ORs) of likelihood of patient satisfaction is based on weighted values

^b^Health Insurance: Only accounted for national health insurance.

^c^Others’ includes Medical Aid class 1 and 2, no health insurance, or unknown.

* p<0.05

## Discussion

Our study revealed that family income, perceived health status, and health insurance status were significant predictors for patient satisfaction with pharmacy services in Korea. With the use of the rich data, findings from our study can provide an important insight on patient satisfaction and its predictors.

One of the major findings in this study indicated that individuals with middle to low family incomes were more likely to be satisfied with pharmacy services. Our results were consistent with the findings from a previous study by Oparah and Kikanme that evaluated consumer satisfaction with pharmacies in a city in Nigeria [19]. They reported that individuals who had lower incomes experienced higher satisfaction toward health services provided [19]. However, Bleichet al. demonstrated that patients with higher income per capita reported higher satisfaction with the health care system in association with more spending on health care expenses and an increased access to advanced technologies [20]. We believe that patient satisfaction in association with financial status can be a more complicated matter and may not be sufficiently explained by family income or spending on health care expenses without considering the insurance structure or health care system of each society.

Interestingly, our study revealed that patients with ‘Fair’ perceived health were more likely to report their satisfaction toward pharmacy services. A number of published studies reported a direct relationship between perceived physical health status and patient satisfaction [21–23]. For example, Rahmqvist reported that patient satisfaction significantly decreased when their subjective health status moved toward ill conditions and pain increased [22]. A study by Xiao and Barber, using the 1999 Medical Expenditure Panel Survey from the United States, reported that patients who rated their health status as excellent or good were more likely to have higher scores on patient satisfaction among all three dimensions of access to care, provider quality, and quality of care [23]. As the measurements of patient satisfaction from KNHANES IV were not specific by service dimensions, patient satisfaction should be cautiously interpreted and further studies are needed to evaluate more accurately the association between perceived health and patient satisfaction with pharmacy services.

In addition, our study showed that insurance type was a significant predictor indicating that individuals insured through their workplace were less likely to be satisfied with pharmacy services. According to the National Health Insurance Corporation in Korea, insured employees contribute 5.08% of their average salary toward the health insurance, while individuals covered through the community based program pay their insurance premium set by primarily their income and assets [24]. The effects of age, perceived health, health status, or economic determinants on pharmacy satisfaction were described in the literature [19–23]. Although higher proportions of the individuals in their 20’s and 30’s are represented in workplace insurance group, the role of health insurance type on pharmacy satisfaction is not reported in the literature and our study is limited on speculating potential reasons for the association. Thus, further studies are in need to describe the impact of health insurance type on their pharmacy satisfaction in Korea.

This study has shown some strength. One of the major changes in the health care system in South Korea was the implementation of the new prescription law in July 2000. Therefore, it has become increasingly important to evaluate patient satisfaction with pharmacy services and to identify subgroups of patients whose needs were not met under the current health care system. However, a literature review showed that published studies on patient satisfaction were mostly from developed countries [16, 25–30] and limited reports are available from Asian countries like South Korea. In this sense, our study used the latest national-level data containing rich information on demographic characteristics, clinical data, and patient-reported health-related outcomes providing a comprehensive picture on the level of patient satisfactions with pharmacy services. From the perspectives of consumers, pharmacists, and policy decision maker, an additional strength of our study is its capacity in offering a balanced view regarding the level of patient satisfaction in South Korea. Moreover, the associated predictors of patient satisfaction was described in our study after adjusting for demographic confounders as well as clinical comorbidity, and thus have a clinical implication in identifying target patient populations for pharmacists to improve the overall level of satisfaction. Evaluations of patient satisfaction with pharmacy services are important efforts in tracking changes in order to prepare structured action plans, to develop innovative pharmacy services, and to conduct program assessments to better serve patients and maximize the professional capacity in pharmacies at local and national levels. From that perspective, we believe continuous collection of patient satisfaction data at a national level would enable researchers and other stakeholders to produce more comprehensive evaluations of pharmacy services, especially in the era of the expanded services by pharmacists in Korea although the pharmacy satisfaction survey component has not been collected after 2008, as part of KNHANES, by KCDC [31].

This study has several limitations. First of all, the data of this survey has more than seven years. However, KNHANES IV data from 2008used in our study was the latest available data source that allowed the evaluation of pharmacy satisfaction at present and pharmacy satisfaction is no longer collected with administrative reasons. Additionally, as this study used the secondary analysis of KNHANES IV, several limitations originating from the nature of the primary dataset should be recognized. Using the KNHANES data, responses to questions of patient satisfaction with pharmacy services relied solely on subjective memory, which may lead to recall bias. However, the bias appeared to be minimal, as the survey asked for patients’ recollection of pharmacy visits made within the last 2 weeks prior to the survey collection. Also the cross-sectional design of the study hinders the identification of the causal relationships between the variables and patient satisfaction. Lastly, this study assessed the global patient satisfaction with pharmacy services. Therefore, the satisfaction level for specific domains of pharmacy services, such as the quality of the counseling, waiting time, physical environment, or drug expenditures, was not considered [4, 21, 32–33]. However, despite its limitations, our study is one of the few studies using national-level data that represent South Korean adult pharmacy users.

## Conclusions

Patients with a fair perception of their health and those with middle to low family incomes were significantly more satisfied with pharmacy services, and employment based health insurers reported significantly lower satisfaction with pharmacy services in South Korea. This implies these patients’ health perception, health insurance type, and the level of family income factors should be taken into consideration concurrently with other predictive determinants when interpreting the results of patient satisfaction to assess the quality of pharmacy services. In an era with the need to improve the quality of health care delivery, continuous evaluation of national level data as a tracking tool can provide important insights in decisions making process from the perspectives of consumers, pharmacists, and policy makers.
